# *μ*-Nitrilo­triacetato-tris(1,10-phenanthroline)dizinc(II) nitrate hexa­hydrate

**DOI:** 10.1107/S1600536808041433

**Published:** 2008-12-10

**Authors:** Lee Fang Chin, Chew Hee Ng, Seik Weng Ng

**Affiliations:** a Faculty of Engineering & Science, Universiti Tunku Abdul Rahman, Jalan Genting Kelang, 53100 Kuala Lumpur, Malaysia; b Department of Chemistry, University of Malaya, 50603 Kuala Lumpur, Malaysia

## Abstract

The nitrilo­triacetate trianion in the title compound, [Zn_2_(C_6_H_6_NO_6_)(C_12_H_8_N_2_)_3_]NO_3_·6H_2_O, is engaged in *N*,*O*,*O*′,*O*"-chelation to the phenanthroline-chelated Zn^II^ unit, giving a distorted octa­hedral geometry for the metal atom. One of the three O atoms of the trianion that is engaged in chelation also binds to the bis­(phenanthroline)-chelated Zn^II^ unit, whose five-coordinate geometry is distorted owing to a long Zn⋯O inter­action [2.401 (2) Å]. The dinuclear cations, nitrate anions and uncoordinated water mol­ecules are linked into a three-dimensional network *via* O—H⋯O hydrogen bonds between water molecules and carboxylate O atoms.

## Related literature

There are no structural examples of *N*-donor ligands of zinc nitrilo­triacetate. For the isostructural copper analog, see: Tang *et al.* (2007 ▶).
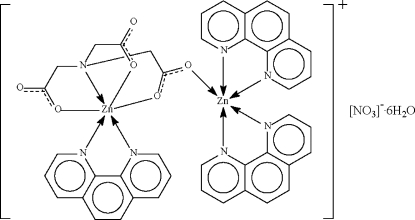


## Experimental

### 

#### Crystal data

[Zn_2_(C_6_H_6_NO_6_)(C_12_H_8_N_2_)_3_]NO_3_·6H_2_O
*M* *_r_* = 1029.58
Monoclinic, 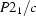

*a* = 12.4729 (2) Å
*b* = 15.2672 (2) Å
*c* = 22.5295 (3) Å
β = 98.678 (1)°
*V* = 4241.1 (1) Å^3^
*Z* = 4
Mo *K*α radiation
μ = 1.21 mm^−1^
*T* = 100 (2) K
0.30 × 0.20 × 0.10 mm

#### Data collection

Bruker SMART APEX diffractometer
Absorption correction: multi-scan (*SADABS*; Sheldrick, 1996 ▶) *T*_min_ = 0.712, *T*_max_ = 0.888
40047 measured reflections
9739 independent reflections
8382 reflections with *I* > 2σ(*I*)
*R*_int_ = 0.024

#### Refinement

*R*[*F* ^2^ > 2σ(*F* ^2^)] = 0.037
*wR*(*F* ^2^) = 0.100
*S* = 1.03
9739 reflections
562 parameters
H-atom parameters constrained
Δρ_max_ = 0.96 e Å^−3^
Δρ_min_ = −0.77 e Å^−3^

### 

Data collection: *APEX2* (Bruker, 2007 ▶); cell refinement: *SAINT* (Bruker, 2007 ▶); data reduction: *SAINT*; program(s) used to solve structure: *SHELXS97* (Sheldrick, 2008 ▶); program(s) used to refine structure: *SHELXL97* (Sheldrick, 2008 ▶); molecular graphics: *X-SEED* (Barbour, 2001 ▶); software used to prepare material for publication: *publCIF* (Westrip, 2009 ▶).

## Supplementary Material

Crystal structure: contains datablocks global, I. DOI: 10.1107/S1600536808041433/ci2741sup1.cif

Structure factors: contains datablocks I. DOI: 10.1107/S1600536808041433/ci2741Isup2.hkl

Additional supplementary materials:  crystallographic information; 3D view; checkCIF report

## Figures and Tables

**Table 1:** Hydrogen-bond geometry (Å, °)

*D*—H⋯*A*	*D*—H	H⋯*A*	*D*⋯*A*	*D*—H⋯*A*
O1*w*—H11⋯O2	0.84	2.03	2.862 (3)	173
O1*w*—H12⋯O2*w*	0.84	1.87	2.706 (3)	179
O2*w*—H21⋯O3*w*	0.84	1.94	2.778 (3)	176
O2*w*—H22⋯O5*w*^i^	0.84	1.92	2.731 (3)	162
O3*w*—H31⋯O9	0.84	2.00	2.803 (3)	160
O3*w*—H32⋯O1*w*^ii^	0.84	1.89	2.732 (3)	173
O4*w*—H41⋯O4	0.84	1.95	2.768 (3)	165
O4*w*—H42⋯O7	0.84	2.16	2.989 (4)	167
O5*w*—H51⋯O6^iii^	0.84	1.95	2.790 (2)	174
O5*w*—H52⋯O4*w*	0.84	1.92	2.750 (3)	173
O6*w*—H61⋯O6	0.85	1.95	2.797 (3)	174
O6*w*—H62⋯O6^iv^	0.85	2.05	2.889 (3)	170

Symmetry codes: (i) 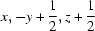; (ii) 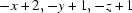; (iii) 
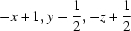
; (iv) 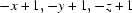.

**Fig. 1.**
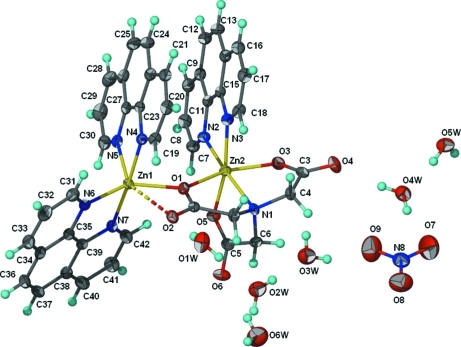
70% Probability displacement ellipsoid plot (Barbour, 2001) of [Zn2(C12H8N2)3(C6H6NO6)](NO3).6H2O. Hydrogen atoms are drawn as spheres of arbitrary radii. The Zn···O interaction of 2.401 (2) Å is shown as a dashed line.

## supplementary crystallographic information

### Experimental

An methanol solution of zinc(II) nitrate hexahydrate (0.30 g, 1 mmol) and 1,10-phenanthroline (0.20 g, 1 mmol) was mixed with an aqueous solution of nitrilotriacetic acid (0.19 g, 1 mmol) and sodium hydroxide 0.12 g, 3 mmol). The mixture was briefly heated. The cool solution yielded a white solid. This was recrystallized from a water-methanol mixture to give colourless crystals.

### Refinement

Carbon-bound H-atoms were placed in calculated positions (C—H 0.95–0.99 Å) and were treated as riding on their parent atoms, with *U*(H) set to 1.2 times *U*_eq_(C). The water H-atoms were placed in chemically sensible positions on the basis of hydrogen bonding, but were not refined; their *U*(H) values were set to 1.5*U*_eq_(O). For the three phenanthroline groups, the central six-membered ring was refined as a rigid hexagon of 1.39 Å sides.

### Crystal data

**Table:**

[Zn_2_(C_6_H_6_NO_6_)(C_12_H_8_N_2_)_3_]NO_3_·6H_2_O	*F*(000) = 2120
*M**_r_* = 1029.58	*D*_x_ = 1.612 Mg m^−^^3^
Monoclinic, *P*2_1_/*c*	Mo *K*α radiation, λ = 0.71073 Å
Hall symbol: -P 2ybc	Cell parameters from 9988 reflections
*a* = 12.4729 (2) Å	θ = 2.4–28.3°
*b* = 15.2672 (2) Å	µ = 1.21 mm^−^^1^
*c* = 22.5295 (3) Å	*T* = 100 K
β = 98.678 (1)°	Block, colourless
*V* = 4241.1 (1) Å^3^	0.30 × 0.20 × 0.10 mm
*Z* = 4	

### Data collection

**Table:**

Bruker SMART APEX diffractometer	9739 independent reflections
Radiation source: fine-focus sealed tube	8382 reflections with *I* > 2σ(*I*)
graphite	*R*_int_ = 0.024
ω scans	θ_max_ = 27.5°, θ_min_ = 1.8°
Absorption correction: multi-scan (*SADABS*; Sheldrick, 1996)	*h* = −16→16
*T*_min_ = 0.712, *T*_max_ = 0.888	*k* = −19→19
40047 measured reflections	*l* = −29→29

### Refinement

**Table:**

Refinement on *F*^2^	Primary atom site location: structure-invariant direct methods
Least-squares matrix: full	Secondary atom site location: difference Fourier map
*R*[*F*^2^ > 2σ(*F*^2^)] = 0.037	Hydrogen site location: inferred from neighbouring sites
*wR*(*F*^2^) = 0.100	H-atom parameters constrained
*S* = 1.03	*w* = 1/[σ^2^(*F*_o_^2^) + (0.0488*P*)^2^ + 6.5337*P*] where *P* = (*F*_o_^2^ + 2*F*_c_^2^)/3
9739 reflections	(Δ/σ)_max_ = 0.001
562 parameters	Δρ_max_ = 0.96 e Å^−^^3^
0 restraints	Δρ_min_ = −0.77 e Å^−^^3^

### Fractional atomic coordinates and isotropic or equivalent isotropic displacement parameters (Å^2^)

**Table:**

	*x*	*y*	*z*	*U*_iso_*/*U*_eq_	
Zn1	0.750335 (19)	0.816173 (16)	0.426850 (11)	0.01349 (7)	
Zn2	0.673278 (19)	0.610471 (16)	0.308663 (11)	0.01354 (7)	
O1	0.72485 (12)	0.68771 (10)	0.38783 (7)	0.0159 (3)	
O2	0.81274 (13)	0.68509 (10)	0.48001 (7)	0.0183 (3)	
O3	0.66383 (13)	0.49744 (11)	0.25608 (7)	0.0183 (3)	
O4	0.74100 (15)	0.36654 (12)	0.25078 (8)	0.0260 (4)	
O5	0.52655 (12)	0.60029 (10)	0.33869 (7)	0.0168 (3)	
O6	0.44311 (13)	0.53088 (12)	0.40537 (7)	0.0219 (4)	
O7	0.8216 (3)	0.07755 (19)	0.36580 (13)	0.0761 (10)	
O8	0.8582 (2)	0.10886 (16)	0.46045 (10)	0.0474 (6)	
O9	0.7954 (2)	0.20621 (17)	0.39637 (11)	0.0552 (7)	
O1W	0.96509 (15)	0.59181 (13)	0.56473 (9)	0.0317 (4)	
H11	0.9165	0.6162	0.5406	0.048*	
H12	0.9456	0.5407	0.5718	0.048*	
O2W	0.90578 (15)	0.42596 (12)	0.58705 (8)	0.0296 (4)	
H21	0.8873	0.4010	0.5538	0.044*	
H22	0.8584	0.4160	0.6091	0.044*	
O3W	0.85426 (17)	0.34521 (14)	0.47592 (8)	0.0345 (4)	
H31	0.8342	0.2971	0.4597	0.052*	
H32	0.9072	0.3642	0.4605	0.052*	
O4W	0.74367 (14)	0.18551 (12)	0.25717 (8)	0.0261 (4)	
H41	0.7502	0.2400	0.2614	0.039*	
H42	0.7604	0.1612	0.2907	0.039*	
O5W	0.73622 (14)	0.13299 (12)	0.13985 (8)	0.0267 (4)	
H51	0.6796	0.1059	0.1256	0.040*	
H52	0.7344	0.1456	0.1758	0.040*	
O6W	0.4344 (2)	0.39461 (15)	0.48746 (10)	0.0490 (6)	
H61	0.4333	0.4339	0.4609	0.073*	
H62	0.4760	0.4114	0.5185	0.073*	
N1	0.72137 (14)	0.50942 (12)	0.37614 (8)	0.0133 (3)	
N2	0.60894 (15)	0.69114 (12)	0.23799 (8)	0.0150 (4)	
N3	0.81863 (15)	0.64807 (12)	0.27339 (8)	0.0154 (4)	
N4	0.90460 (15)	0.82165 (12)	0.39941 (8)	0.0140 (3)	
N5	0.71238 (15)	0.88131 (12)	0.34426 (8)	0.0155 (4)	
N6	0.77717 (15)	0.89515 (12)	0.50346 (8)	0.0153 (4)	
N7	0.59262 (15)	0.81921 (12)	0.45128 (8)	0.0156 (4)	
N8	0.82499 (19)	0.12888 (16)	0.40803 (10)	0.0295 (5)	
C1	0.77454 (17)	0.64633 (14)	0.43311 (10)	0.0145 (4)	
C2	0.79166 (18)	0.54821 (14)	0.42784 (10)	0.0163 (4)	
H2A	0.8684	0.5370	0.4238	0.020*	
H2B	0.7769	0.5193	0.4651	0.020*	
C3	0.72086 (18)	0.43336 (15)	0.27824 (10)	0.0170 (4)	
C4	0.77312 (18)	0.44007 (14)	0.34455 (10)	0.0157 (4)	
H4A	0.7659	0.3832	0.3647	0.019*	
H4B	0.8514	0.4530	0.3468	0.019*	
C5	0.52224 (17)	0.54273 (15)	0.37824 (10)	0.0159 (4)	
C6	0.61716 (18)	0.47836 (15)	0.39219 (10)	0.0168 (4)	
H6A	0.6277	0.4651	0.4357	0.020*	
H6B	0.5972	0.4230	0.3705	0.020*	
C7	0.50481 (18)	0.71028 (15)	0.22108 (10)	0.0181 (4)	
H7	0.4541	0.6947	0.2468	0.022*	
C8	0.46713 (18)	0.75248 (15)	0.16680 (10)	0.0191 (4)	
H8	0.3922	0.7652	0.1561	0.023*	
C9	0.53936 (19)	0.77523 (15)	0.12924 (10)	0.0182 (4)	
H9	0.5149	0.8040	0.0923	0.022*	
C11	0.68324 (9)	0.71289 (9)	0.20064 (6)	0.0151 (4)	
C10	0.65230 (8)	0.75528 (10)	0.14615 (6)	0.0169 (4)	
C12	0.72919 (11)	0.77442 (10)	0.10935 (5)	0.0198 (5)	
H12A	0.7080	0.8034	0.0721	0.024*	
C13	0.83702 (10)	0.75116 (10)	0.12705 (6)	0.0208 (5)	
H13	0.8896	0.7642	0.1019	0.025*	
C14	0.86796 (8)	0.70877 (10)	0.18155 (6)	0.0174 (4)	
C15	0.79107 (10)	0.68964 (9)	0.21834 (5)	0.0148 (4)	
C16	0.97822 (19)	0.68396 (15)	0.20203 (11)	0.0209 (5)	
H16	1.0329	0.6952	0.1779	0.025*	
C17	1.00403 (18)	0.64399 (15)	0.25654 (11)	0.0204 (5)	
H17	1.0769	0.6278	0.2709	0.024*	
C18	0.92185 (18)	0.62707 (15)	0.29118 (11)	0.0187 (4)	
H18	0.9409	0.5994	0.3291	0.022*	
C19	0.99819 (18)	0.78963 (15)	0.42686 (10)	0.0171 (4)	
H19	1.0014	0.7668	0.4663	0.020*	
C20	1.09217 (18)	0.78818 (15)	0.39994 (11)	0.0196 (5)	
H20	1.1576	0.7644	0.4209	0.023*	
C21	1.08920 (18)	0.82129 (15)	0.34310 (11)	0.0190 (4)	
H21A	1.1521	0.8196	0.3240	0.023*	
C23	0.89980 (10)	0.85564 (9)	0.34208 (5)	0.0147 (4)	
C22	0.99012 (8)	0.85846 (9)	0.31275 (6)	0.0176 (4)	
C24	0.98156 (9)	0.89386 (10)	0.25539 (6)	0.0219 (5)	
H24	1.0433	0.8958	0.2353	0.026*	
C25	0.88269 (10)	0.92642 (9)	0.22735 (5)	0.0225 (5)	
H25	0.8768	0.9506	0.1881	0.027*	
C26	0.79237 (8)	0.92359 (7)	0.25667 (5)	0.0189 (4)	
C27	0.80092 (8)	0.88820 (8)	0.31404 (5)	0.0153 (4)	
C28	0.68803 (10)	0.95545 (11)	0.22961 (7)	0.0233 (5)	
H28	0.6788	0.9806	0.1906	0.028*	
C29	0.6018 (2)	0.94917 (16)	0.26064 (12)	0.0247 (5)	
H29	0.5323	0.9703	0.2435	0.030*	
C30	0.61725 (19)	0.91133 (15)	0.31788 (11)	0.0194 (4)	
H30	0.5568	0.9070	0.3388	0.023*	
C31	0.87041 (19)	0.92828 (15)	0.53061 (11)	0.0195 (5)	
H31A	0.9337	0.9203	0.5124	0.023*	
C32	0.8793 (2)	0.97433 (16)	0.58478 (11)	0.0231 (5)	
H32A	0.9476	0.9960	0.6033	0.028*	
C33	0.7882 (2)	0.98778 (15)	0.61090 (11)	0.0213 (5)	
H33	0.7931	1.0187	0.6478	0.026*	
C35	0.68454 (8)	0.90717 (9)	0.52979 (6)	0.0158 (4)	
C34	0.68572 (9)	0.95506 (9)	0.58236 (6)	0.0182 (4)	
C36	0.59038 (11)	0.96703 (9)	0.60630 (5)	0.0207 (5)	
H36	0.5912	0.9998	0.6422	0.025*	
C37	0.49385 (9)	0.93110 (9)	0.57767 (5)	0.0209 (5)	
H37	0.4287	0.9393	0.5940	0.025*	
C38	0.49266 (8)	0.88321 (7)	0.52510 (5)	0.0181 (4)	
C39	0.58800 (9)	0.87124 (8)	0.50116 (5)	0.0157 (4)	
C40	0.39636 (9)	0.84143 (11)	0.49471 (7)	0.0220 (5)	
H40	0.3290	0.8489	0.5089	0.026*	
C41	0.40267 (19)	0.79078 (17)	0.44505 (12)	0.0236 (5)	
H41A	0.3397	0.7629	0.4246	0.028*	
C42	0.50283 (18)	0.78049 (15)	0.42477 (11)	0.0195 (5)	
H42A	0.5065	0.7444	0.3908	0.023*	

### Atomic displacement parameters (Å^2^)

**Table:**

	*U*^11^	*U*^22^	*U*^33^	*U*^12^	*U*^13^	*U*^23^
Zn1	0.01251 (12)	0.01347 (13)	0.01506 (13)	0.00027 (9)	0.00391 (9)	−0.00111 (9)
Zn2	0.01236 (12)	0.01544 (13)	0.01283 (12)	0.00060 (9)	0.00198 (9)	0.00228 (9)
O1	0.0156 (7)	0.0135 (7)	0.0187 (8)	0.0013 (6)	0.0032 (6)	0.0011 (6)
O2	0.0191 (8)	0.0181 (8)	0.0181 (8)	−0.0024 (6)	0.0041 (6)	−0.0054 (6)
O3	0.0201 (8)	0.0205 (8)	0.0138 (7)	−0.0001 (6)	0.0011 (6)	−0.0012 (6)
O4	0.0311 (10)	0.0252 (9)	0.0221 (9)	0.0042 (7)	0.0054 (7)	−0.0083 (7)
O5	0.0132 (7)	0.0205 (8)	0.0169 (8)	0.0015 (6)	0.0025 (6)	0.0030 (6)
O6	0.0154 (8)	0.0326 (9)	0.0185 (8)	0.0005 (7)	0.0053 (6)	0.0046 (7)
O7	0.124 (3)	0.0513 (17)	0.0581 (17)	−0.0281 (17)	0.0291 (18)	−0.0287 (14)
O8	0.0474 (14)	0.0583 (15)	0.0353 (12)	0.0080 (11)	0.0028 (10)	0.0141 (11)
O9	0.0716 (18)	0.0452 (14)	0.0434 (14)	0.0175 (13)	−0.0092 (12)	0.0040 (11)
O1W	0.0277 (10)	0.0265 (10)	0.0376 (11)	0.0031 (8)	−0.0054 (8)	−0.0026 (8)
O2W	0.0313 (10)	0.0310 (10)	0.0262 (9)	−0.0033 (8)	0.0036 (8)	0.0029 (8)
O3W	0.0446 (12)	0.0342 (11)	0.0252 (10)	0.0038 (9)	0.0067 (8)	0.0046 (8)
O4W	0.0225 (9)	0.0295 (10)	0.0264 (9)	0.0010 (7)	0.0042 (7)	−0.0018 (7)
O5W	0.0221 (9)	0.0335 (10)	0.0243 (9)	−0.0070 (7)	0.0032 (7)	0.0007 (7)
O6W	0.0672 (16)	0.0452 (13)	0.0335 (12)	−0.0131 (12)	0.0046 (11)	−0.0019 (10)
N1	0.0130 (8)	0.0140 (8)	0.0126 (8)	0.0009 (7)	0.0013 (7)	−0.0027 (7)
N2	0.0127 (8)	0.0169 (9)	0.0155 (9)	0.0007 (7)	0.0027 (7)	0.0021 (7)
N3	0.0136 (9)	0.0165 (9)	0.0160 (9)	0.0005 (7)	0.0016 (7)	0.0015 (7)
N4	0.0136 (8)	0.0136 (8)	0.0147 (9)	−0.0003 (7)	0.0021 (7)	−0.0003 (7)
N5	0.0149 (9)	0.0132 (8)	0.0183 (9)	0.0005 (7)	0.0016 (7)	−0.0004 (7)
N6	0.0165 (9)	0.0135 (9)	0.0162 (9)	−0.0007 (7)	0.0034 (7)	0.0004 (7)
N7	0.0150 (9)	0.0147 (9)	0.0176 (9)	−0.0003 (7)	0.0039 (7)	−0.0025 (7)
N8	0.0272 (11)	0.0340 (12)	0.0286 (12)	−0.0088 (9)	0.0081 (9)	−0.0050 (10)
C1	0.0111 (9)	0.0150 (10)	0.0187 (10)	−0.0017 (8)	0.0061 (8)	−0.0008 (8)
C2	0.0187 (10)	0.0148 (10)	0.0140 (10)	0.0013 (8)	−0.0018 (8)	−0.0016 (8)
C3	0.0150 (10)	0.0212 (11)	0.0157 (10)	−0.0034 (8)	0.0060 (8)	−0.0028 (8)
C4	0.0168 (10)	0.0142 (10)	0.0161 (10)	0.0022 (8)	0.0020 (8)	−0.0022 (8)
C5	0.0143 (10)	0.0197 (11)	0.0133 (10)	−0.0027 (8)	0.0008 (8)	−0.0028 (8)
C6	0.0179 (10)	0.0165 (10)	0.0168 (10)	0.0001 (8)	0.0053 (8)	0.0032 (8)
C7	0.0157 (10)	0.0194 (11)	0.0198 (11)	0.0016 (8)	0.0047 (8)	0.0022 (9)
C8	0.0147 (10)	0.0205 (11)	0.0211 (11)	0.0029 (8)	−0.0002 (8)	0.0014 (9)
C9	0.0209 (11)	0.0167 (10)	0.0160 (10)	0.0032 (8)	−0.0002 (8)	0.0011 (8)
C11	0.0173 (10)	0.0120 (9)	0.0166 (10)	−0.0018 (8)	0.0043 (8)	−0.0009 (8)
C10	0.0188 (11)	0.0150 (10)	0.0169 (10)	0.0005 (8)	0.0025 (8)	−0.0007 (8)
C12	0.0252 (12)	0.0190 (11)	0.0154 (10)	0.0003 (9)	0.0041 (9)	0.0023 (8)
C13	0.0225 (11)	0.0206 (11)	0.0214 (11)	−0.0024 (9)	0.0102 (9)	0.0013 (9)
C14	0.0167 (10)	0.0154 (10)	0.0209 (11)	−0.0020 (8)	0.0049 (8)	−0.0017 (8)
C15	0.0165 (10)	0.0121 (10)	0.0163 (10)	−0.0008 (8)	0.0038 (8)	−0.0008 (8)
C16	0.0160 (11)	0.0199 (11)	0.0281 (12)	−0.0020 (9)	0.0076 (9)	−0.0020 (9)
C17	0.0130 (10)	0.0208 (11)	0.0271 (12)	0.0003 (9)	0.0022 (9)	0.0002 (9)
C18	0.0147 (10)	0.0175 (11)	0.0232 (11)	−0.0008 (8)	0.0003 (8)	0.0024 (9)
C19	0.0159 (10)	0.0163 (10)	0.0186 (11)	−0.0004 (8)	0.0011 (8)	0.0011 (8)
C20	0.0117 (10)	0.0179 (11)	0.0288 (12)	0.0008 (8)	0.0021 (9)	−0.0005 (9)
C21	0.0154 (10)	0.0168 (10)	0.0265 (12)	−0.0022 (8)	0.0082 (9)	−0.0018 (9)
C23	0.0175 (10)	0.0122 (9)	0.0149 (10)	−0.0030 (8)	0.0040 (8)	−0.0025 (8)
C22	0.0176 (11)	0.0149 (10)	0.0209 (11)	−0.0038 (8)	0.0046 (8)	−0.0028 (8)
C24	0.0241 (12)	0.0209 (12)	0.0230 (12)	−0.0047 (9)	0.0104 (9)	−0.0005 (9)
C25	0.0312 (13)	0.0198 (11)	0.0165 (11)	−0.0048 (10)	0.0041 (9)	0.0022 (9)
C26	0.0240 (12)	0.0135 (10)	0.0184 (11)	−0.0014 (9)	0.0005 (9)	0.0006 (8)
C27	0.0182 (10)	0.0112 (9)	0.0164 (10)	−0.0014 (8)	0.0023 (8)	−0.0023 (8)
C28	0.0293 (13)	0.0195 (11)	0.0188 (11)	−0.0002 (10)	−0.0041 (9)	0.0048 (9)
C29	0.0205 (11)	0.0223 (12)	0.0285 (13)	0.0045 (9)	−0.0056 (9)	0.0026 (10)
C30	0.0173 (11)	0.0178 (11)	0.0227 (11)	0.0026 (9)	0.0019 (9)	0.0000 (9)
C31	0.0184 (11)	0.0181 (11)	0.0221 (11)	−0.0022 (9)	0.0030 (9)	0.0007 (9)
C32	0.0222 (12)	0.0205 (11)	0.0249 (12)	−0.0061 (9)	−0.0020 (9)	−0.0019 (9)
C33	0.0292 (12)	0.0174 (11)	0.0168 (11)	−0.0030 (9)	0.0016 (9)	−0.0032 (9)
C35	0.0185 (11)	0.0131 (10)	0.0161 (10)	0.0033 (8)	0.0035 (8)	0.0023 (8)
C34	0.0248 (12)	0.0123 (10)	0.0174 (11)	0.0009 (8)	0.0028 (9)	0.0010 (8)
C36	0.0292 (12)	0.0170 (11)	0.0166 (11)	0.0035 (9)	0.0055 (9)	−0.0021 (9)
C37	0.0230 (12)	0.0216 (11)	0.0205 (11)	0.0049 (9)	0.0105 (9)	0.0008 (9)
C38	0.0189 (11)	0.0157 (10)	0.0205 (11)	0.0027 (8)	0.0056 (9)	0.0019 (8)
C39	0.0193 (11)	0.0140 (10)	0.0145 (10)	0.0015 (8)	0.0046 (8)	0.0019 (8)
C40	0.0155 (11)	0.0259 (12)	0.0258 (12)	0.0014 (9)	0.0073 (9)	−0.0018 (10)
C41	0.0141 (11)	0.0268 (12)	0.0301 (13)	−0.0036 (9)	0.0036 (9)	−0.0046 (10)
C42	0.0179 (11)	0.0206 (11)	0.0204 (11)	−0.0004 (9)	0.0037 (9)	−0.0049 (9)

### Geometric parameters (Å, °)

**Table:**

Zn1—N6	2.0907 (19)	C8—C9	1.370 (3)
Zn1—N5	2.0999 (19)	C8—H8	0.95
Zn1—N4	2.1096 (18)	C9—C10	1.435 (2)
Zn1—N7	2.1217 (19)	C9—H9	0.95
Zn1—O1	2.1532 (15)	C11—C10	1.39
Zn1—O2	2.4008 (16)	C11—C15	1.39
Zn2—O5	2.0506 (15)	C10—C12	1.39
Zn2—N2	2.0761 (18)	C12—C13	1.39
Zn2—O3	2.0864 (16)	C12—H12A	0.95
Zn2—O1	2.1539 (16)	C13—C14	1.39
Zn2—N3	2.1639 (18)	C13—H13	0.95
Zn2—N1	2.1853 (18)	C14—C15	1.39
O1—C1	1.278 (3)	C14—C16	1.434 (3)
O2—C1	1.242 (3)	C16—C17	1.366 (3)
O3—C3	1.267 (3)	C16—H16	0.95
O4—C3	1.238 (3)	C17—C18	1.403 (3)
O5—C5	1.258 (3)	C17—H17	0.95
O6—C5	1.250 (3)	C18—H18	0.95
O7—N8	1.228 (3)	C19—C20	1.399 (3)
O8—N8	1.230 (3)	C19—H19	0.95
O9—N8	1.253 (3)	C20—C21	1.372 (3)
O1W—H11	0.84	C20—H20	0.95
O1W—H12	0.84	C21—C22	1.436 (3)
O2W—H21	0.84	C21—H21A	0.95
O2W—H22	0.84	C23—C22	1.39
O3W—H31	0.84	C23—C27	1.39
O3W—H32	0.84	C22—C24	1.39
O4W—H41	0.84	C24—C25	1.39
O4W—H42	0.84	C24—H24	0.95
O5W—H51	0.84	C25—C26	1.39
O5W—H52	0.84	C25—H25	0.95
O6W—H61	0.85	C26—C27	1.39
O6W—H62	0.85	C26—C28	1.436
N1—C2	1.472 (3)	C28—C29	1.372 (3)
N1—C4	1.477 (3)	C28—H28	0.95
N1—C6	1.479 (3)	C29—C30	1.400 (3)
N2—C7	1.330 (3)	C29—H29	0.95
N2—C11	1.383 (2)	C30—H30	0.95
N3—C18	1.329 (3)	C31—C32	1.398 (3)
N3—C15	1.389 (2)	C31—H31A	0.95
N4—C19	1.329 (3)	C32—C33	1.371 (3)
N4—C23	1.385 (2)	C32—H32A	0.95
N5—C30	1.326 (3)	C33—C34	1.432 (3)
N5—C27	1.386 (2)	C33—H33	0.95
N6—C31	1.330 (3)	C35—C34	1.39
N6—C35	1.388 (2)	C35—C39	1.39
N7—C42	1.326 (3)	C34—C36	1.39
N7—C39	1.385 (2)	C36—C37	1.39
C1—C2	1.520 (3)	C36—H36	0.95
C2—H2A	0.99	C37—C38	1.39
C2—H2B	0.99	C37—H37	0.95
C3—C4	1.541 (3)	C38—C39	1.39
C4—H4A	0.99	C38—C40	1.4389
C4—H4B	0.99	C40—C41	1.372 (3)
C5—C6	1.534 (3)	C40—H40	0.95
C6—H6A	0.99	C41—C42	1.402 (3)
C6—H6B	0.99	C41—H41A	0.95
C7—C8	1.399 (3)	C42—H42A	0.95
C7—H7	0.95		
			
N6—Zn1—N5	116.43 (7)	C8—C9—C10	119.52 (19)
N6—Zn1—N4	100.35 (7)	C8—C9—H9	120.2
N5—Zn1—N4	79.35 (7)	C10—C9—H9	120.2
N6—Zn1—N7	79.19 (7)	N2—C11—C10	121.81 (11)
N5—Zn1—N7	97.22 (7)	N2—C11—C15	118.18 (11)
N4—Zn1—N7	175.95 (7)	C10—C11—C15	120.0
N6—Zn1—O1	149.07 (7)	C11—C10—C12	120.0
N5—Zn1—O1	94.01 (6)	C11—C10—C9	117.62 (13)
N4—Zn1—O1	90.08 (6)	C12—C10—C9	122.35 (13)
N7—Zn1—O1	92.31 (6)	C13—C12—C10	120.0
N6—Zn1—O2	93.98 (6)	C13—C12—H12A	120.0
N5—Zn1—O2	148.16 (6)	C10—C12—H12A	120.0
N4—Zn1—O2	86.43 (6)	C12—C13—C14	120.0
N7—Zn1—O2	97.61 (6)	C12—C13—H13	120.0
O1—Zn1—O2	57.39 (6)	C14—C13—H13	120.0
O5—Zn2—N2	92.12 (7)	C15—C14—C13	120.0
O5—Zn2—O3	98.50 (6)	C15—C14—C16	117.84 (13)
N2—Zn2—O3	94.19 (7)	C13—C14—C16	122.16 (13)
O5—Zn2—O1	86.45 (6)	N3—C15—C14	121.89 (11)
N2—Zn2—O1	110.05 (7)	N3—C15—C11	118.11 (11)
O3—Zn2—O1	155.12 (6)	C14—C15—C11	120.0
O5—Zn2—N3	168.57 (7)	C17—C16—C14	119.5 (2)
N2—Zn2—N3	79.01 (7)	C17—C16—H16	120.3
O3—Zn2—N3	89.40 (7)	C14—C16—H16	120.3
O1—Zn2—N3	89.82 (6)	C16—C17—C18	119.3 (2)
O5—Zn2—N1	83.22 (6)	C16—C17—H17	120.3
N2—Zn2—N1	170.41 (7)	C18—C17—H17	120.3
O3—Zn2—N1	78.30 (6)	N3—C18—C17	122.9 (2)
O1—Zn2—N1	78.12 (6)	N3—C18—H18	118.5
N3—Zn2—N1	106.59 (7)	C17—C18—H18	118.5
C1—O1—Zn1	95.76 (13)	N4—C19—C20	122.6 (2)
C1—O1—Zn2	116.02 (13)	N4—C19—H19	118.7
Zn1—O1—Zn2	147.57 (8)	C20—C19—H19	118.7
C1—O2—Zn1	85.35 (13)	C21—C20—C19	119.5 (2)
C3—O3—Zn2	115.77 (14)	C21—C20—H20	120.3
C5—O5—Zn2	114.97 (14)	C19—C20—H20	120.3
H11—O1W—H12	109.6	C20—C21—C22	119.35 (19)
H21—O2W—H22	108.8	C20—C21—H21A	120.3
H31—O3W—H32	108.8	C22—C21—H21A	120.3
H41—O4W—H42	109.2	N4—C23—C22	122.07 (11)
H51—O5W—H52	109.8	N4—C23—C27	117.93 (11)
H61—O6W—H62	108.2	C22—C23—C27	120.0
C2—N1—C4	114.71 (17)	C23—C22—C24	120.0
C2—N1—C6	112.08 (17)	C23—C22—C21	117.54 (12)
C4—N1—C6	110.61 (17)	C24—C22—C21	122.43 (12)
C2—N1—Zn2	109.55 (13)	C22—C24—C25	120.0
C4—N1—Zn2	105.36 (13)	C22—C24—H24	120.0
C6—N1—Zn2	103.73 (13)	C25—C24—H24	120.0
C7—N2—C11	119.21 (17)	C26—C25—C24	120.0
C7—N2—Zn2	126.73 (15)	C26—C25—H25	120.0
C11—N2—Zn2	113.09 (12)	C24—C25—H25	120.0
C18—N3—C15	118.52 (18)	C25—C26—C27	120.0
C18—N3—Zn2	130.74 (16)	C25—C26—C28	122.4
C15—N3—Zn2	109.93 (12)	C27—C26—C28	117.6
C19—N4—C23	118.90 (17)	N5—C27—C26	122.08 (10)
C19—N4—Zn1	128.68 (15)	N5—C27—C23	117.88 (10)
C23—N4—Zn1	112.04 (12)	C26—C27—C23	120.0
C30—N5—C27	118.63 (18)	C29—C28—C26	119.35 (12)
C30—N5—Zn1	128.87 (16)	C29—C28—H28	120.3
C27—N5—Zn1	112.42 (12)	C26—C28—H28	120.3
C31—N6—C35	118.67 (18)	C28—C29—C30	119.2 (2)
C31—N6—Zn1	128.25 (16)	C28—C29—H29	120.4
C35—N6—Zn1	112.90 (12)	C30—C29—H29	120.4
C42—N7—C39	119.06 (17)	N5—C30—C29	123.0 (2)
C42—N7—Zn1	128.79 (15)	N5—C30—H30	118.5
C39—N7—Zn1	112.14 (12)	C29—C30—H30	118.5
O7—N8—O8	123.5 (3)	N6—C31—C32	122.8 (2)
O7—N8—O9	117.7 (3)	N6—C31—H31A	118.6
O8—N8—O9	118.8 (2)	C32—C31—H31A	118.6
O2—C1—O1	121.4 (2)	C33—C32—C31	119.2 (2)
O2—C1—C2	119.7 (2)	C33—C32—H32A	120.4
O1—C1—C2	118.79 (19)	C31—C32—H32A	120.4
N1—C2—C1	112.60 (18)	C32—C33—C34	119.8 (2)
N1—C2—H2A	109.1	C32—C33—H33	120.1
C1—C2—H2A	109.1	C34—C33—H33	120.1
N1—C2—H2B	109.1	N6—C35—C34	122.07 (11)
C1—C2—H2B	109.1	N6—C35—C39	117.91 (11)
H2A—C2—H2B	107.8	C34—C35—C39	120.0
O4—C3—O3	125.7 (2)	C36—C34—C35	120.0
O4—C3—C4	116.8 (2)	C36—C34—C33	122.55 (12)
O3—C3—C4	117.45 (19)	C35—C34—C33	117.41 (12)
N1—C4—C3	111.29 (17)	C37—C36—C34	120.0
N1—C4—H4A	109.4	C37—C36—H36	120.0
C3—C4—H4A	109.4	C34—C36—H36	120.0
N1—C4—H4B	109.4	C36—C37—C38	120.0
C3—C4—H4B	109.4	C36—C37—H37	120.0
H4A—C4—H4B	108.0	C38—C37—H37	120.0
O6—C5—O5	124.7 (2)	C39—C38—C37	120.0
O6—C5—C6	116.67 (19)	C39—C38—C40	117.3
O5—C5—C6	118.58 (19)	C37—C38—C40	122.7
N1—C6—C5	115.09 (18)	N7—C39—C38	122.22 (10)
N1—C6—H6A	108.5	N7—C39—C35	117.63 (10)
C5—C6—H6A	108.5	C38—C39—C35	120.0
N1—C6—H6B	108.5	C41—C40—C38	119.44 (12)
C5—C6—H6B	108.5	C41—C40—H40	120.3
H6A—C6—H6B	107.5	C38—C40—H40	120.3
N2—C7—C8	122.4 (2)	C40—C41—C42	119.5 (2)
N2—C7—H7	118.8	C40—C41—H41A	120.2
C8—C7—H7	118.8	C42—C41—H41A	120.2
C9—C8—C7	119.4 (2)	N7—C42—C41	122.5 (2)
C9—C8—H8	120.3	N7—C42—H42A	118.8
C7—C8—H8	120.3	C41—C42—H42A	118.8
			
N6—Zn1—O1—C1	−26.4 (2)	Zn2—O5—C5—O6	174.35 (17)
N5—Zn1—O1—C1	163.52 (13)	Zn2—O5—C5—C6	−9.5 (2)
N4—Zn1—O1—C1	84.20 (13)	C2—N1—C6—C5	96.4 (2)
N7—Zn1—O1—C1	−99.07 (13)	C4—N1—C6—C5	−134.18 (19)
O2—Zn1—O1—C1	−1.52 (11)	Zn2—N1—C6—C5	−21.6 (2)
N6—Zn1—O1—Zn2	164.81 (12)	O6—C5—C6—N1	−160.66 (19)
N5—Zn1—O1—Zn2	−5.26 (15)	O5—C5—C6—N1	22.8 (3)
N4—Zn1—O1—Zn2	−84.59 (15)	C11—N2—C7—C8	−0.5 (3)
N7—Zn1—O1—Zn2	92.14 (15)	Zn2—N2—C7—C8	−168.38 (17)
O2—Zn1—O1—Zn2	−170.30 (17)	N2—C7—C8—C9	0.0 (4)
O5—Zn2—O1—C1	94.85 (15)	C7—C8—C9—C10	0.1 (3)
N2—Zn2—O1—C1	−174.18 (14)	C7—N2—C11—C10	0.8 (2)
O3—Zn2—O1—C1	−7.8 (2)	Zn2—N2—C11—C10	170.29 (8)
N3—Zn2—O1—C1	−95.96 (15)	C7—N2—C11—C15	−177.90 (16)
N1—Zn2—O1—C1	11.04 (14)	Zn2—N2—C11—C15	−8.44 (16)
O5—Zn2—O1—Zn1	−97.59 (15)	N2—C11—C10—C12	−178.71 (16)
N2—Zn2—O1—Zn1	−6.62 (16)	C15—C11—C10—C12	0.0
O3—Zn2—O1—Zn1	159.79 (12)	N2—C11—C10—C9	−0.67 (18)
N3—Zn2—O1—Zn1	71.60 (15)	C15—C11—C10—C9	178.04 (16)
N1—Zn2—O1—Zn1	178.60 (16)	C8—C9—C10—C11	0.2 (3)
N6—Zn1—O2—C1	169.03 (13)	C8—C9—C10—C12	178.18 (17)
N5—Zn1—O2—C1	−27.66 (19)	C11—C10—C12—C13	0.0
N4—Zn1—O2—C1	−90.83 (13)	C9—C10—C12—C13	−177.94 (17)
N7—Zn1—O2—C1	89.42 (13)	C10—C12—C13—C14	0.0
O1—Zn1—O2—C1	1.56 (12)	C12—C13—C14—C15	0.0
O5—Zn2—O3—C3	−104.52 (15)	C12—C13—C14—C16	−179.56 (17)
N2—Zn2—O3—C3	162.70 (15)	C18—N3—C15—C14	0.7 (2)
O1—Zn2—O3—C3	−4.5 (2)	Zn2—N3—C15—C14	−170.11 (8)
N3—Zn2—O3—C3	83.77 (15)	C18—N3—C15—C11	−178.76 (16)
N1—Zn2—O3—C3	−23.31 (15)	Zn2—N3—C15—C11	10.47 (16)
N2—Zn2—O5—C5	168.84 (16)	C13—C14—C15—N3	−179.41 (16)
O3—Zn2—O5—C5	74.29 (16)	C16—C14—C15—N3	0.16 (18)
O1—Zn2—O5—C5	−81.19 (15)	C13—C14—C15—C11	0.0
N3—Zn2—O5—C5	−152.4 (3)	C16—C14—C15—C11	179.57 (16)
N1—Zn2—O5—C5	−2.75 (15)	N2—C11—C15—N3	−1.81 (17)
O5—Zn2—N1—C2	−106.36 (14)	C10—C11—C15—N3	179.44 (15)
O3—Zn2—N1—C2	153.47 (14)	N2—C11—C15—C14	178.75 (15)
O1—Zn2—N1—C2	−18.57 (13)	C10—C11—C15—C14	0.0
N3—Zn2—N1—C2	67.64 (14)	C15—C14—C16—C17	−0.8 (3)
O5—Zn2—N1—C4	129.76 (13)	C13—C14—C16—C17	178.72 (17)
O3—Zn2—N1—C4	29.58 (13)	C14—C16—C17—C18	0.7 (3)
O1—Zn2—N1—C4	−142.46 (13)	C15—N3—C18—C17	−0.8 (3)
N3—Zn2—N1—C4	−56.25 (14)	Zn2—N3—C18—C17	167.69 (17)
O5—Zn2—N1—C6	13.47 (13)	C16—C17—C18—N3	0.2 (4)
O3—Zn2—N1—C6	−86.71 (13)	C23—N4—C19—C20	−0.5 (3)
O1—Zn2—N1—C6	101.26 (13)	Zn1—N4—C19—C20	171.66 (16)
N3—Zn2—N1—C6	−172.53 (13)	N4—C19—C20—C21	0.4 (3)
O5—Zn2—N2—C7	−8.24 (19)	C19—C20—C21—C22	1.2 (3)
O3—Zn2—N2—C7	90.45 (19)	C19—N4—C23—C22	−1.0 (2)
O1—Zn2—N2—C7	−95.24 (19)	Zn1—N4—C23—C22	−174.41 (8)
N3—Zn2—N2—C7	179.0 (2)	C19—N4—C23—C27	179.55 (15)
O5—Zn2—N2—C11	−176.74 (13)	Zn1—N4—C23—C27	6.10 (15)
O3—Zn2—N2—C11	−78.06 (13)	N4—C23—C22—C24	−179.48 (15)
O1—Zn2—N2—C11	96.26 (13)	C27—C23—C22—C24	0.0
N3—Zn2—N2—C11	10.52 (13)	N4—C23—C22—C21	2.51 (18)
O5—Zn2—N3—C18	140.0 (3)	C27—C23—C22—C21	−178.01 (14)
N2—Zn2—N3—C18	179.6 (2)	C20—C21—C22—C23	−2.6 (3)
O3—Zn2—N3—C18	−86.0 (2)	C20—C21—C22—C24	179.42 (16)
O1—Zn2—N3—C18	69.1 (2)	C23—C22—C24—C25	0.0
N1—Zn2—N3—C18	−8.4 (2)	C21—C22—C24—C25	177.91 (15)
O5—Zn2—N3—C15	−50.8 (4)	C22—C24—C25—C26	0.0
N2—Zn2—N3—C15	−11.14 (12)	C24—C25—C26—C27	0.0
O3—Zn2—N3—C15	83.24 (13)	C24—C25—C26—C28	−179.3
O1—Zn2—N3—C15	−121.62 (13)	C30—N5—C27—C26	−1.5 (2)
N1—Zn2—N3—C15	160.84 (12)	Zn1—N5—C27—C26	175.52 (8)
N6—Zn1—N4—C19	66.7 (2)	C30—N5—C27—C23	−179.38 (15)
N5—Zn1—N4—C19	−178.1 (2)	Zn1—N5—C27—C23	−2.34 (14)
O1—Zn1—N4—C19	−84.07 (19)	C25—C26—C27—N5	−177.82 (15)
O2—Zn1—N4—C19	−26.75 (19)	C28—C26—C27—N5	1.56 (15)
N6—Zn1—N4—C23	−120.70 (13)	C25—C26—C27—C23	0.0
N5—Zn1—N4—C23	−5.48 (12)	C28—C26—C27—C23	179.4
O1—Zn1—N4—C23	88.58 (13)	N4—C23—C27—N5	−2.60 (14)
O2—Zn1—N4—C23	145.90 (13)	C22—C23—C27—N5	177.91 (14)
N6—Zn1—N5—C30	−82.8 (2)	N4—C23—C27—C26	179.50 (15)
N4—Zn1—N5—C30	−179.1 (2)	C22—C23—C27—C26	0.0
N7—Zn1—N5—C30	−1.3 (2)	C25—C26—C28—C29	178.78 (17)
O1—Zn1—N5—C30	91.5 (2)	C27—C26—C28—C29	−0.58 (17)
O2—Zn1—N5—C30	115.9 (2)	C26—C28—C29—C30	−0.4 (3)
N6—Zn1—N5—C27	100.57 (13)	C27—N5—C30—C29	0.5 (3)
N4—Zn1—N5—C27	4.21 (12)	Zn1—N5—C30—C29	−176.00 (18)
N7—Zn1—N5—C27	−177.95 (12)	C28—C29—C30—N5	0.5 (4)
O1—Zn1—N5—C27	−85.12 (12)	C35—N6—C31—C32	0.4 (3)
O2—Zn1—N5—C27	−60.77 (19)	Zn1—N6—C31—C32	−174.42 (17)
N5—Zn1—N6—C31	−91.3 (2)	N6—C31—C32—C33	−1.3 (4)
N4—Zn1—N6—C31	−8.2 (2)	C31—C32—C33—C34	−0.3 (3)
N7—Zn1—N6—C31	175.9 (2)	C31—N6—C35—C34	2.2 (2)
O1—Zn1—N6—C31	99.7 (2)	Zn1—N6—C35—C34	177.75 (8)
O2—Zn1—N6—C31	78.92 (19)	C31—N6—C35—C39	−179.28 (15)
N5—Zn1—N6—C35	93.61 (14)	Zn1—N6—C35—C39	−3.71 (15)
N4—Zn1—N6—C35	176.77 (12)	N6—C35—C34—C36	178.51 (15)
N7—Zn1—N6—C35	0.87 (13)	C39—C35—C34—C36	0.0
O1—Zn1—N6—C35	−75.31 (19)	N6—C35—C34—C33	−3.65 (18)
O2—Zn1—N6—C35	−96.13 (13)	C39—C35—C34—C33	177.84 (15)
N6—Zn1—N7—C42	−179.5 (2)	C32—C33—C34—C36	−179.55 (17)
N5—Zn1—N7—C42	64.8 (2)	C32—C33—C34—C35	2.7 (3)
O1—Zn1—N7—C42	−29.5 (2)	C35—C34—C36—C37	0.0
O2—Zn1—N7—C42	−86.9 (2)	C33—C34—C36—C37	−177.73 (15)
N6—Zn1—N7—C39	1.96 (12)	C34—C36—C37—C38	0.0
N5—Zn1—N7—C39	−113.67 (13)	C36—C37—C38—C39	0.0
O1—Zn1—N7—C39	152.00 (13)	C36—C37—C38—C40	177.4
O2—Zn1—N7—C39	94.59 (13)	C42—N7—C39—C38	1.1 (2)
Zn1—O2—C1—O1	−2.59 (19)	Zn1—N7—C39—C38	179.74 (8)
Zn1—O2—C1—C2	174.31 (18)	C42—N7—C39—C35	176.69 (16)
Zn1—O1—C1—O2	2.9 (2)	Zn1—N7—C39—C35	−4.64 (15)
Zn2—O1—C1—O2	176.23 (15)	C37—C38—C39—N7	175.52 (15)
Zn1—O1—C1—C2	−174.03 (16)	C40—C38—C39—N7	−2.04 (15)
Zn2—O1—C1—C2	−0.7 (2)	C37—C38—C39—C35	0.0
C4—N1—C2—C1	141.89 (18)	C40—C38—C39—C35	−177.6
C6—N1—C2—C1	−90.9 (2)	N6—C35—C39—N7	5.71 (15)
Zn2—N1—C2—C1	23.7 (2)	C34—C35—C39—N7	−175.72 (14)
O2—C1—C2—N1	166.81 (19)	N6—C35—C39—C38	−178.57 (15)
O1—C1—C2—N1	−16.2 (3)	C34—C35—C39—C38	0.0
Zn2—O3—C3—O4	−167.15 (19)	C39—C38—C40—C41	1.44 (17)
Zn2—O3—C3—C4	10.7 (2)	C37—C38—C40—C41	−176.05 (17)
C2—N1—C4—C3	−153.33 (18)	C38—C40—C41—C42	0.1 (3)
C6—N1—C4—C3	78.7 (2)	C39—N7—C42—C41	0.6 (3)
Zn2—N1—C4—C3	−32.78 (19)	Zn1—N7—C42—C41	−177.86 (18)
O4—C3—C4—N1	−165.09 (19)	C40—C41—C42—N7	−1.1 (4)
O3—C3—C4—N1	16.9 (3)		

### Hydrogen-bond geometry (Å, °)

**Table:**

*D*—H···*A*	*D*—H	H···*A*	*D*···*A*	*D*—H···*A*
O1w—H11···O2	0.84	2.03	2.862 (3)	173
O1w—H12···O2w	0.84	1.87	2.706 (3)	179
O2w—H21···O3w	0.84	1.94	2.778 (3)	176
O2w—H22···O5w^i^	0.84	1.92	2.731 (3)	162
O3w—H31···O9	0.84	2.00	2.803 (3)	160
O3w—H32···O1w^ii^	0.84	1.89	2.732 (3)	173
O4w—H41···O4	0.84	1.95	2.768 (3)	165
O4w—H42···O7	0.84	2.16	2.989 (4)	167
O5w—H51···O6^iii^	0.84	1.95	2.790 (2)	174
O5w—H52···O4w	0.84	1.92	2.750 (3)	173
O6w—H61···O6	0.85	1.95	2.797 (3)	174
O6w—H62···O6^iv^	0.85	2.05	2.889 (3)	170

Symmetry codes: (i) *x*, −*y*+1/2, *z*+1/2; (ii) −*x*+2, −*y*+1, −*z*+1; (iii) −*x*+1, *y*−1/2, −*z*+1/2; (iv) −*x*+1, −*y*+1, −*z*+1.

## References

[bb7] Barbour, L. J. (2001). *J. Supramol. Chem.* **1**, 189–191.

[bb1] Bruker (2007). *APEX2* and *SAINT* Bruker AXS Inc., Madison, Wisconsin, USA.

[bb3] Sheldrick, G. M. (1996). *SADABS* University of Göttingen, Germany.

[bb4] Sheldrick, G. M. (2008). *Acta Cryst.* A**64**, 112–122. 10.1107/S0108767307043930 18156677

[bb5] Tang, X., Liang, F., Chen, J., Li, Y., Xu, Y. & Shen, W. (2007). *Mater. Chem. Phys.* **106**, 159–163.

[bb6] Westrip, S. P. (2009). *publCIF* In preparation.

